# The effect of zoledronic acid on type and volume of Modic changes among patients with low back pain

**DOI:** 10.1186/s12891-017-1632-z

**Published:** 2017-06-23

**Authors:** Katri Koivisto, Jyri Järvinen, Jaro Karppinen, Marianne Haapea, Markus Paananen, Eero Kyllönen, Osmo Tervonen, Jaakko Niinimäki

**Affiliations:** 1 Medical Research Center Oulu, Oulu University Hospital and University of Oulu, Oulu, Finland; 2 Institute of Diagnostics, Department of Diagnostic Radiology, Oulu University Hospital, Oulu, Finland; 3 Center for Life Course Health Research, University of Oulu, Oulu, Finland; 4 Finnish Institute of Occupational Health, Oulu, Finland

**Keywords:** Magnetic resonance imaging, Modic changes, Type of Modic changes, Randomized trial, Zoledronic acid, Low back pain

## Abstract

**Background:**

Modic changes (MC) are associated with low back pain (LBP). In this study, we compared changes in size and type of MC, after a single intravenous infusion of 5 mg zoledronic acid (ZA) or placebo, among chronic LBP patients with MC on magnetic resonance imaging (MRI), and evaluated whether the MRI changes correlate with symptoms.

**Methods:**

All patients (*N* = 19 in ZA, 20 in placebo) had MRI at baseline (0.23–1.5 T) and at one year (1.5-3 T). We evaluated the level, type and volume of all the MC. The MC were classified into M1 (M1 (100%)), predominating M1 (M1/2 (65:35%)) or predominating M2 (M1/2 (35:65%)), and M2 (M2 (100%)). The first two were considered M1-dominant, and the latter two M2-dominant. Volumes of M1 and M2 were calculated separately for the primary MC, which was assumed to cause the symptoms, and the other MC. We analysed the one-year treatment differences in M1 and M2 volumes using analysis of covariance with adjustments for age, sex, body mass index, and smoking. The correlations between the MRI changes and the changes in LBP symptoms were analysed using Pearson correlations.

**Results:**

In the ZA group, 84.2% of patients had M1-dominant primary MC at baseline, compared to 50% in the placebo group (*p* = 0.041). The primary MC in the ZA group converted more likely to M2-dominant (42.1% ZA, 15% placebo; *p* = 0.0119). The other MC (15 ZA, 8 placebo) were on average 42% smaller and remained largely M2-dominant. The M1 volume of the primary MC decreased in the ZA group, but increased in the placebo group (−0.83 cm^3^ vs 0.91 cm^3^; *p* = 0.21). The adjusted treatment difference for M1 volume was −1.9 cm^3^ (95% CI -5.0 to 1.2; *p* = 0.22) and for M2 volume 0.23 cm^3^ (*p* = 0.86). In the MC that remained M1-dominant, volume change correlated positively with increased symptoms in the placebo group, whereas the correlations were negative and weak in the ZA group.

**Conclusions:**

Zoledronic acid tended to speed up the conversion of M1-dominant into M2-dominant MC and decrease the volume of M1-dominant MC, although statistical significance was not demonstrated.

**Trial registration:**

The registration number in ClinicalTrials.gov is NCT01330238 and the date of registration February 11, 2011.

## Background

Modic changes (MC) are vertebral endplate and bone marrow changes that are visible on magnetic resonance imaging (MRI). Three different types of MC are described: Type 1 MC (M1) show fibrovascular replacement of bone marrow and are considered to be the earliest stage in the process of MC evolution, representing an inflammatory lesion; Type 2 MC (M2) show fatty replacement of the red bone marrow; while Type 3 MC (M3) are associated with subchondral bone sclerosis [1–4]. The identification of mixed types (M1/2 and M2/3) is thought to indicate different stages of the same pathologic process, as MC are able to convert from one type to another [5–8].

According to a systematic review, MC are more common among patients with low back pain (LBP) than among asymptomatic volunteers [9]. Many studies note that M1 are associated more strongly with LBP than with other MC types [10, 11]. In a Finnish study, the conversion of M1 to M2 over two years was associated with improvement of pain intensity and disability [12]. Similar results were also obtained in a Danish study, where the presence of M1 at both baseline and 14-month follow-up were associated with poor outcomes among patients with persistent LBP and MC [13].

We have previously shown that a single intravenous infusion of 5 mg zoledronic acid (ZA) reduces the intensity of LBP in the short term, compared to placebo [14]. We hypothesize that the beneficial effect of ZA on symptoms may be due to the conversion of M1 to M2. Therefore, the objective of our study was to compare the effect of a single intravenous infusion of 5 mg ZA to that of a placebo infusion on the change in size and type of MC, and to determine whether this change correlates with improvement in clinical symptoms.

## Methods

### Study population

The study population consisted of patients with chronic LBP and MC on MRI [14]. Inclusion criteria were LBP for at least three months, LBP intensity of at least six on a 10-cm Visual Analog Scale (VAS) or an Oswestry Disability Index (ODI) [15] of at least 30%, and an MC on MRI performed within no more than six months prior to enrolment. The exclusion criteria included renal impairment, hypoalcaemia, hypersensitivity to bisphosphonates or the infusion, the presence of red flags, nerve root entrapment, willingness for early retirement, and childbearing potential [14].

The Oulu University Hospital ethics committee approved the study protocol. All patients provided written informed consent before any study-specific procedures were performed. This study was registered (ClinicalTrials.gov, unique identifier NCT01330238) and was conducted in accordance with the principles of the Declaration of Helsinki.

### Treatment intervention

Patients were randomized to receive a single intravenous infusion of 5 mg ZA in 100 ml saline (*N* = 19) or 100 ml saline as placebo (*N* = 20) over a 15-min period. Before administration of the infusion, all patients received 600 mg oral ibuprofen or 1 g paracetamol to prevent acute phase reactions, and 100,000 units of Vitamin D (Vigantol®) to prevent hypocalcaemia. The patients, the principal investigator performing the screening and follow-up assessments, and the radiologist evaluating the MRI scans were blinded to the treatment allocation.

### Magnetic resonance imaging

Baseline imaging was performed on average four months (standard deviation (SD) 3 months, range 0.4 to 11.5 months) before the infusion. Follow-up scans were obtained on average 11.9 months (SD 0.6, range 11 to 13 months) after the infusion, with an average 15.9-month interval (SD 3.2, range 12.1 to 23.5 months) between them. Baseline MRIs were performed in the district of the Oulu University Hospital with five 1.5 T units (GE Signa Twinspeed, General Electric Medical Systems, Milwaukee, WI, USA; Philips Achieva and Philips Intera, Philips Medical Systems, Eindhoven, The Netherlands; Siemens Avanto and Siemens Espree, Siemens Medical, Erlangen, Germany), a 0.34 T unit (Siemens Magnetom C, Siemens Medical, Erlangen, Germany) and a 0.23 T unit (Philips Panorama, Philips Medical Systems, Eindhoven, The Netherlands). Imaging protocols varied somewhat due to the multiple units used. The protocols were of clinical imaging purpose established to spine imaging. The imaging parameters of the sagittal T1-weighted (T1 W) turbo spin-echo (TSE) or fast spin-echo (FSE) sequences with fluid attenuation inversion recovery (FLAIR) were repetition time (TR) 1800–2270 ms/inversion time (TI) 860 ms/echo time (TE) 9–29 ms (*N* = 16), and without FLAIR: TR 326–793 ms/TE 8–18 ms (*N* = 23). The imaging parameters of the sagittal T2-weighted (T2 W) TSE/FSE sequences were TR 3000–4500 ms/TE 105–130 ms (*N* = 39). The imaging parameters of the short tau inversion recovery sequences (STIR) were, for example, TR 3400/ TI 150/ TE 70. The spacing, including slice thickness and slice gap, was 4.4–6.2 mm in all sequences.

At one-year follow-up, MRIs were performed with two 1.5 T units (GE Signa Twinspeed and GE Optima, General Electric Medical Systems, Milwaukee, WI, USA) and a 3 T unit (Siemens Skyra, Siemens Medical, Erlangen, Germany). The imaging parameters of the sagittal T1-weighted TSE or FSE sequences with FLAIR were TR 2047–2270 ms/TI 860-900 ms/TE 9–29 ms (*N* = 37) and without FLAIR: TR 540–587/TE 12–24 ms (*N* = 2). The imaging parameters of the sagittal T2-weighted TSE or FSE sequences were TR 2796–3500 ms/TE 101–123 ms (*N* = 39). Spacing was 3.6–5 mm in all sequences.

### Image analysis

MRIs were analysed for type and volume of each MC from sagittal images taken by a Fellow in musculoskeletal radiology (JJ) at a clinical workstation (Neaview Radiology, version 2.23, Neagen Corporation, Finland). In order to examine the interobserver reliability, an experienced musculoskeletal radiologist (JN) analysed the images of 19 randomly selected patients.

MC type was assessed using T1 W and T2 W images, and the MRI scans were classified as previously described [11]: M1 showed low signal intensity (SI) on T1 W and high SI on T2 W and STIR images, M2 showed high SI on both T1 W and T2 W images and low SI on STIR images, and M3 showed low SI on both T1 W and T2 W images.

MC type was divided into four groups: M1 (100%), predominating M1 (M1/2 (65:35%)), predominating M2 (M1/2 (35:65%)) and M2 (100%). The first two were considered M1- dominant, and the latter two M2-dominant. M1 and M2 were defined as consisting totally of oedemic or fatty signal changes, respectively; whereas predominating M1 and predominating M2 were defined as mixed Type 1/2 MC with more oedemic or fatty signal changes, respectively. The classification was data driven. The proportion of M3 was so low that it was excluded from the analyses. The area (cm^2^) of the MC was measured slice by slice from T2 W images by a workstation area tool. The volume (cm^3^) of the MC was calculated by multiplying the area with the spacing.

Since some individuals had multiple MC, a primary MC was defined to represent the most likely LBP generator. The severity of the lesion was assumed as follows: M1 > predominating M1 > predominating M2 > M2. In cases when patients with multiple MC had the same types at different levels, the larger MC was selected as the primary MC. The characteristics of the primary MC and other MC were evaluated separately.

Interobserver reliability was substantial for raw MC type classification (M1, predominating M1, predominating M2 and M2; linearly weighted kappa 0.65); also for dichotomized data (M1-dominant vs. M2-dominant; kappa 0.73). The reliability of the volume measurements was almost perfect (intraclass correlation coefficient 0.92).

### Statistical analysis

Baseline characteristics were described using frequencies with proportions, mean values with SD or median values with interquartile ranges, separately for ZA and placebo groups. Cross tabulations were used to describe the distributions of MC type in the ZA and placebo groups at baseline and at one year. The treatment groups’ MC type at baseline and their change in MC type during the follow-up were compared using the Chi square test. The treatment groups’ mean MC volumes at baseline and the changes in their MC volumes were compared using the t-test. In addition, the changes in MC volumes were compared adjusted for age, sex, body mass index, and smoking, using analysis of covariance. The correlations between the changes in MC volumes and the changes in intensity of LBP and ODI were analysed using Pearson correlations. Interobserver reliability was analysed using Cohen’s kappa for dichotomized, and linearly weighted kappa for raw, MC type classification, and intraclass correlation coefficient for the volume of MC. They were all interpreted as follows: 0.00–0.20 slight, 0.21–0.40 fair, 0.41–0.60 moderate, 0.61–0.80 substantial, 0.81–1.00 almost perfect [16].

## Results

### Study population

All 39 enrolled, eligible patients completed the one-year follow-up. The ZA and placebo groups were similar in clinical characteristics at baseline (Table 1). The patients’ mean age was 50 and mean BMI 26.8. The median duration of LBP was 315 days, and the mean VAS score for LBP was 6.6.

**Table 1:** Baseline characteristics of the study population according to treatment group

Characteristics	Total	Zoledronic acid *n* = 19	Placebo *n* = 20
Sex, *n* (%) men	25 (64.1)	14 (73.7)	11 (55.0)
Age, mean (SD) years	50.4 (8.4)	49.4 (9.5)	51.5 (7.3)
Smoking, *n* (%) regular smokers^a^	11 (28.2)	5 (26.3)	6 (30.0)
BMI, mean (SD) kg/m	26.8 (3.2)	26.2 (3.3)	27.4 (3.2)
Duration of LBP, median (IQ range) days	315 (212, 365)	330 (200, 365)	300 (270, 365)
Intensity of LBP, mean (SD)^b^	6.6 (1.5)	6.5 (1.4)	6.8 (1.6)
Oswestry Disability Index, mean % (SD)	32.9 (10.4)	30.7 (10.9)	34.9 (9.8)
Duration of sick leave during the past year, median (IQ range) days	20 (0, 65)	20 (0, 50)	18 (1, 181)

*BMI* Body Mass Index, *LBP* low back pain, *SD* standard deviation, *IQ* inter-quartile

^a^Smoking at least one cigarette/day

^b^Assessed using a 10-cm Visual Analogue Scale (VAS)

#### MRI findings

**Fig. 1:**
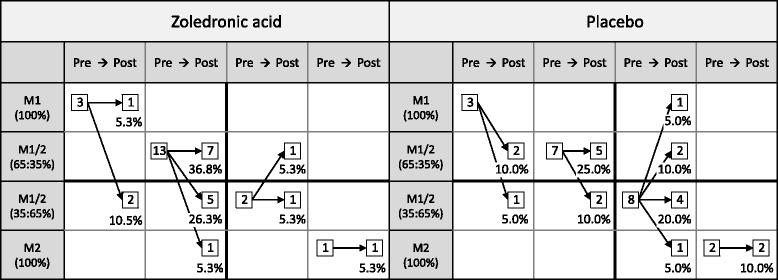
Course of MC types of primary MC during one-year follow-up. *Arrows* indicate the change of MC type (in percent)

**Fig. 2:**
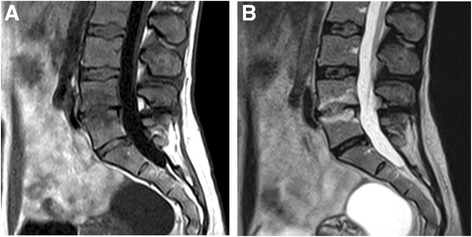
Baseline scans of a 46-year-old female using 0.34 T scanner. **a** T1- and **b** T2- weighted images show M1 at L4/5 level

**Fig. 3:**
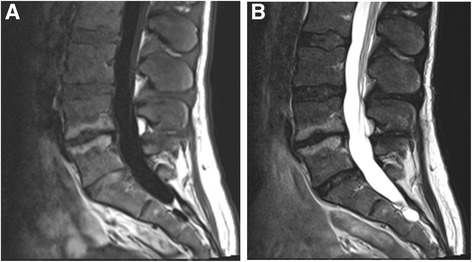
Follow-up scans of the same female than in Fig. 2 using 1.5 T scanner. **a** T1- and **b** T2- weighted images show M2 at L4/5 level

**Fig. 4:**
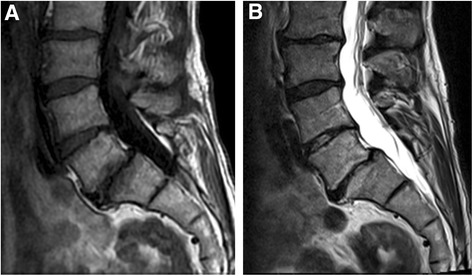
Baseline scans of a 56-year-old male using 1.5 T scanner. **a** T1- and **b** T2- weighted images show M1 at L5/S1 level

**Fig. 5:**
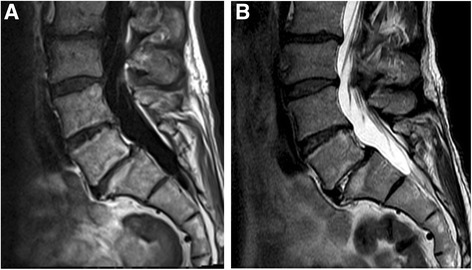
Follow-up scans of the same male than in Fig. 4 using 1.5 T scanner. **a** T1- and **b** T2- weighted images show M2 at L5/S1 level

Primary MC occurred most commonly (71.8%**)** at L4/5 and L5/S1 (Table 2). At baseline, 6 (15.4%) patients had M1, 20 (51.3%) had predominating M1, 10 (25.6%) had predominating M2, and 3 (7.7%) had M2 (Fig. 1). The total volume of MC was 11.4 cm^3^ at baseline and 13.6 cm^3^ at one year. M1-dominant MC were more common in the ZA group (*n* = 16, 84.2%) than in the placebo group (*n* = 10, 50.0%; *p* = 0.041). In the ZA group, eight (42.1%) M1-dominant MC converted to M2-dominant and in the placebo group, only three (15.0%) (*p* = 0.119; Fig. 1). Two examples of MC converting from M1 to M2 in the ZA group are shown in Figs. 2, 3, 4 and 5.

**Table 2:** Level and volume of the primary Modic change at baseline and follow-up and the change in the volume according to treatment group

Volume of the primary Modic change^a^	Mean (SD)		Mean (SD) change			Age-adjusted analyses		Adjusted analyses	
	ZA *n* = 19	Placebo *n* = 20	ZA	Placebo	P^b^	Difference (95% CI)	P^c^	Difference (95% CI)	P^d^
Level^a^, n (%)									
L2/3	4 (21.1)	0 (0.0)							
L3/4	2 (10.5)	5 (25.0)							
L4/5	6 (31.6)	5 (25.0)							
L5/S1	7 (36.8)	10 (50.0)							
Volume of M1^a^ (cm^3^)									
Baseline	7.37 (4.58)	5.04 (3.55)							
Follow-up	6.54 (5.38)	5.95 (4.46)	-0.83 (4.44)	0.91 (4.02)	0.21	−1.79 (−4.58, 1.00)	0.20	−1.92 (−5.03, 1.19)	0.22
Volume of M2^a^ (cm^3^)									
Baseline	4.53 (4.00)	5.87 (4.84)							
Follow-up	6.94 (4.57)	7.84 (6.78)	2.40 (2.95)	1.97 (4.20)	0.71	0.34 (−2.07, 2.74)	0.78	0.232 (−2.44, 2.90)	0.86
Total volume^a^ (cm^3^)									
Baseline	11.90 (5.27)	10.91 (5.96)							
Follow-up	13.48 (5.55)	13.79 (6.64)	1.58 (2.24)	2.88 (3.92)	0.21	−1.45 (−3.55, 0.64)	0.17	−1.69 (−4.14, 0.76)	0.17

*SD* standard deviation, *CI* confidence interval, *ZA* zoledronic acid

^a^The primary Modic change was assumed to cause patients’ symptoms at baseline

^b^Change in the volume compared between the treatment groups, significance from the independent samples t-test

^c^Analysis of covariance for change in the volume compared between the treatment groups, adjusted for age

^d^Analysis of covariance for change in the volume compared between the treatment groups, adjusted for age, sex, Body Mass Index, and smoking

The total volume (ZA and placebo groups) of the primary MC at baseline was 8.3 cm^3^ for M1, 11.3 cm^3^ for predominating M1, 12.5 cm^3^ for predominating M2, and 14.3 cm^3^ for M2. The total volume of the ZA and placebo groups’ primary MC did not differ at baseline (11.9 cm^3^ vs. 10.9 cm^3^, *p* = 0.59; Table 2). The total volume of the primary MC increased from baseline to one year by 1.6 cm^3^ in the ZA group, in comparison to 2.9 cm^3^ in the placebo group (*p* = 0.21; Table 2).

The change in M1 volume was larger in the placebo group than that in the ZA group (0.91 cm^3^ (increase) vs. −0.83 cm^3^ (decrease); *p* = 0.21). In the ZA group, the M1 volume decreased by 11% whereas in the placebo group, it increased by 18%. The change in M2 volume was similar in both groups (1.97 cm^3^ placebo vs. 2.40 cm^3^ ZA; *p* = 0.71). M2 volume increased by 53% in the ZA group and by 34% in the placebo group.

Other MC (15 ZA, 8 placebo) were on average 42% smaller in size than the primary MC (7.0 cm^3^ vs 11.4 cm^3^; Table 3). Of these, 14 (93.3%) were M2-dominant in the ZA group and six (75.0%) in the placebo group. The majority of M2-dominant MC did not convert (12 ZA, 3 placebo) over the follow-up period. The total volume increased by 5.1% in the ZA group and 11% in the placebo group (Table 3). The proportion of M1 volume of the total volume was 10% in the ZA group and 22% in the placebo group at baseline, and 21% and 36%, respectively, at one year.

**Table 3:** Number, level and volume of other Modic changes (MC) than the primary Modic change^a^ at baseline and follow-up and the change in the volume according to treatment group

Volume of other than the primary Modic change^a^	Mean (SD)		Mean (SD) change		Unadjusted analyses	
	ZA	Placebo	ZA	Placebo	Difference (95% CI)	P^b^
Multiple MCs, *n* (%)						
At two levels	9 (60)	6 (75)				
At three levels	6 (40)	2 (25)				
Level of the other than the primary MC, n (%)						
L1/2	1 (6.7)	0 (0.0)				
L2/3	1 (6.7)	1 (12.5)				
L3/4	2 (13.3)	0 (0.0)				
L4/5	5 (33.3)	4 (50.0)				
L5/S1	6 (40.0)	3 (37.5)				
Volume of M1^a^ (cm^3^)						
Baseline	0.80 (1.92)	1.20 (1.62)				
Follow-up	1.69 (2.50)	2.22 (2.20)	0.89 (2.23)	1.03 (2.21)	−0.14 (−2.16, 1.89)	0.89
Volume of M2^a^ (cm^3^)						
Baseline	6.98 (8.01)	4.36 (5.27)				
Follow-up	6.49 (6.74)	3.93 (4.72)	−0.49 (2.54)	−0.43 (1.49)	−0.06 (−2.10, 1.98)	0.95
Total volume^a^ (cm^3^)						
Baseline	7.78 (8.49)	5.55 (5.36)				
Follow-up	8.18 (7.75)	6.15 (5.24)	0.40 (1.09)	0.60 (1.53)	−0.19 (−1.33, 0.95)	0.73

*SD* standard deviation, *CI* confidence interval, *ZA* zoledronic acid

^a^The primary Modic change was assumed to cause patients’ symptoms at baseline

^b^Independent samples t-test for change in the volume between the treatment groups

#### MRI findings and LBP

The overall change in the primary MC volume did not correlate with the change in intensity of LBP or ODI (Pearson’s correlations (r) 0.11 and 0.07, respectively). The volume change in the MC that stayed M1- dominant over the follow-up period correlated positively with increased LBP intensity and ODI in the placebo group (*r* = 0.81 and 0.58, respectively), whereas the corresponding correlations were negative and weak in the ZA group (*r* = −0.21 and −0.28, respectively). In the placebo group, the volume change in the MC that changed from M2- dominant to M1-dominant correlated positively with the change in intensity of LBP and ODI (*r* = 0.70 and 0.89, respectively). The corresponding correlations were negative in the three MC that changed from M1-dominant to M2-dominant (*r* = −0.72 and −0.98, respectively).

**Fig. 6:**
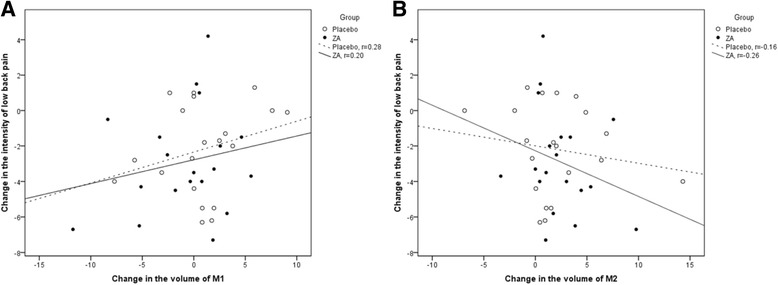
Scatter plots showing **a** the positive correlation between change in M1 volume and change in LBP intensity and **b** the negative correlation between change in M2 volume and change in LBP intensity

**Fig. 7:**
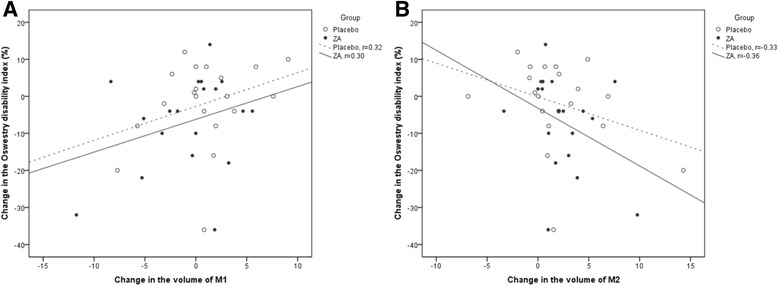
Scatter plots showing **a** the positive correlation between change in M1 volume and change in the Oswestry Disability Index and **b** the negative correlation between change in M2 volume and change in the Oswestry Disability Index

The changes in M1 and M2 volumes in relation to intensity of LBP and ODI are presented in Figs. 6 and 7. Although the correlations were weak, they were consistent with the correlations of the MC that remained either M1-dominant or M2-dominant over the follow-up.

## Discussion

The single intravenous infusion of 5 mg ZA more likely led to conversion of M1-dominant primary MC to M2-dominant than the placebo infusion (in ZA group 42.1% vs. placebo group 15%). The total MC volume increased in both groups, but in the ZA group, the volume of M1 decreased by 11%, whereas in the placebo group the M1 volume increased by 18%. In the MC that remained M1- dominant over the follow-up period, the volume change correlated positively with the increased LBP intensity and ODI in the placebo group, whereas the correlations were negative and weak in the ZA group.

In the present study, the primary MC of the majority of the patients was of a mixed-type (76.9%) and only a minority (15.4%) had M1. In a study of 64 chronic LBP patients, the distribution of MC types was quite similar; 79% had M1/2 and 21% M1 [17]. In a study of 1142 subjects from the general population, 24.7% had MC, 7.1% M1 and 17.6% M2 [18]. It has been suggested that M1 represents the major transition point from normality, and should therefore be the focus when evaluating the relevance of MC [19].

In the placebo group of the current study, only three (15%) M1-dominant MC converted to M2-dominant MC, and the total volume of the primary MC increased by 26%. Of the MC types, the M1 volume increased by 18% and M2 volume by 34%. In a one to six-year follow-up study of 48 M1 among LBP patients, 37.5% of MC totally converted to M2, 14.6% partially to M2, 39.6% remained as M1 but became more extensive, while 8.3% showed no change [20]. In a three-year follow-up study, 10 of 70 discs (14%) with MC baseline converted to another type [6]. In a study of 64 chronic LBP patients, the mean size of MC in relation to vertebrae size was 21% at baseline and 24% at follow-up, and the proportion of the M1 component of the MC decreased from 74 to 41% over two years [12]. In our study, the follow-up period was only one year, which may explain the slower conversion rate of M1-dominant to M2-dominant MC, and the smaller proportion of M2 at follow-up than that in a study by Mitra et al. (12.5 vs 37.5) [20].

In the ZA group, eight (42.1%) M1-dominant MC converted to M2-dominant MC, the total volume of primary MC increased by 13%, and M2 volume by 53%, while M1 volume decreased by 13%. In a Danish study with a 14-month follow-up, LBP intensity among patients who had M1 at both baseline and follow-up was less likely to improve, but change in size of M1 did not correlate with change in LBP intensity [13]. The present study showed a trend of a more likely conversion from M1 to M2 in the ZA group, as 42.1% of M1-dominant MC converted to M2-dominant MC in the ZA group as opposed to only 15% in the placebo group. Our preliminary hypothesis, that ZA might speed up the natural course of MC by enhancing the conversion from M1 to M2, was thus supported by these results. The more likely conversion from M1 to M2 in the ZA group may well be interpreted as a healing process in the natural course of MC. Thus, a single infusion of ZA seemed to positively influence the natural course of MC.

In the present study, overall change in MC volume did not correlate with the change in LBP symptoms. In the placebo group, we observed an increase in symptoms for MC that remained M1 dominant over the follow-up, whereas in the ZA group the corresponding correlations were negative and weak, indicating a slight improvement in symptoms. In a study of 64 chronic LBP patients, change in the size of M1 associated positively with changes in LBP symptoms [12]. The current results in the placebo group support the findings of Järvinen et al. [12], whereas the ZA group obtained almost opposite results. Previously, ZA had reduced the progression of bone oedema on MRI among patients with psoriatic arthritis [21], improved knee symptoms and reduced bone marrow lesion size among patients with knee osteoarthritis [22].

The present study demonstrated that a single infusion of ZA speeds up the evolution of M1-dominant MC toward M2-dominant MC, and decreases the size of M1. The influence of bisphosphonates remains unclear, but bisphosphonates have several potential mechanisms. The pathological process of MC is characterized by inflammation, high bone turnover and fibrosis [23]. The chemical and mechanical stimulation of the nociceptors adjacent to damaged endplates is probably the source of pain. Growing evidence shows that as well as affecting osteoclasts, bisphosphonates also exert effects on osteoblasts, osteocytes and adipocytes [24–26], which might explain the positive effects of ZA in this study.

The strength of our study is its randomized study design. Further strengths include complete follow-up with no drop-outs and 100% adherence, as the medication was given intravenously. Moreover, the radiologist was blinded to the treatment allocation and an experienced musculoskeletal radiologist analysed images of 19 randomly selected patients. We had substantial interobserver reliability, showing that our classification of MC is repeatable.

However, our study had some limitations. At baseline, MRI was performed with different MRI equipment, because M1-dominant MC are not common, and patients were referred to our clinic from several health care units. This variability in MRI equipment reflects clinical practice. The differences between the field strengths of different MRI equipment (0.23–1.5 T) may have some influence on the detection and classification of MC [27]. However, our sensitivity analysis using only high-field MRI scanners did not have significant effect on the results. Variation in the time between baseline imaging and the infusion ranged from 0.4 to 11.5 months, the mean being four months. Five patients clearly had a longer period from baseline to infusion, 9.1–11.5 months. They all had mixed-type primary MC (placebo group, two predominating M2 and one predominating M1; and ZA group, two predominating M1). As the conversion of MC to another type is a long process [2] we do not expect that the long interval before treatment markedly influenced the course of MC.

Our sample size is rather small for detecting changes in MC phenotypes. We did not perform a priori power calculations due to a lack of any previous data on the efficacy of ZA in the investigated indication. A longer follow-up period was not considered necessary for the current study because the response of ZA was expected within one year. We estimated the effect of ZA separately for primary MC, which is assumed to cause patients symptoms, and other than primary MC, because we assumed that they represent different stages of the disease process and behave in a different way. The former were mostly M1-dominant and we expected that the medication would decrease inflammation. We did not expect dramatic changes in other MC, as they were smaller in size and mostly M2-dominant. A Danish study has shown that small MC, observed in the endplates only, are more likely to change size over time or even normalize, whereas larger MC are more likely to persist [7].

## Conclusions

In conclusion, this randomized, placebo-controlled, double-blinded trial showed that the MC in the ZA group tended to convert from M1-dominant MC to M2-dominant MC, and the M1 volume tended to decrease, although statistical significance was not demonstrated. Even though the results are interesting, future studies with a standardized MRI protocol using a single field strength and a larger sample size are required to demonstrate the efficacy of ZA on changes of MC phenotypes.

## Abbreviations

BMI: Body mass index
FLAIR: Fluid attenuation inversion recovery
FSE: Fast spin-echo
IQ: Inter-quartile
LBP: Low back pain
MC: Modic change
MRI: Magnetic resonance imaging
ODI: Oswestry disability index
SD: Standard deviation
STIR: Short tau inversion recovery sequences
T1 W: T1-weighted
T2 W: T2-weighted
TE: Echo time
TI: Inversion time
TR: Repetition time
TSE: Turbo spin-echo
VAS: Visual analog scale
ZA: Zoledronic acid
